# Modulating lysosomal function through lysosome membrane permeabilization or autophagy suppression restores sensitivity to cisplatin in refractory non-small-cell lung cancer cells

**DOI:** 10.1371/journal.pone.0184922

**Published:** 2017-09-25

**Authors:** Magdalena Circu, James Cardelli, Martin Barr, Kenneth O’Byrne, Glenn Mills, Hazem El-Osta

**Affiliations:** 1 Feist-Weiller Cancer Center, Louisiana State University Health Sciences Center-Shreveport, Shreveport, Louisiana, United States of America; 2 Segue Therapeutics, LLC, Shreveport, Louisiana, United States of America; 3 Thoracic Oncology Research Group, Trinity Translational Medicine Institute, Trinity Centre for Health Sciences, St. James’s Hospital & Trinity College Dublin, Dublin, Ireland; 4 Cancer & Ageing Research Program, Queensland University of Technology, Brisbane, Australia; Univerzitet u Beogradu, SERBIA

## Abstract

Lung cancer is the leading cause of cancer-related deaths. Most patients develop resistance to platinum within several months of treatment. We investigated whether triggering lysosomal membrane permeabilization (LMP) or suppressing autophagy can restore cisplatin susceptibility in lung cancer with acquired chemoresistance. Cisplatin IC_50_ in A549Pt (parental) and A549cisR (cisplatin resistant) cells was 13 μM and 47 μM, respectively. Following cisplatin exposure, A549cisR cells failed to elicit an apoptotic response. This was manifested by diminished Annexin–V staining, caspase 3 and 9, BAX and BAK activation in resistant but not in parental cells. Chloroquine preferentially promoted LMP in A549cisR cells, revealed by leakage of FITC-dextran into the cytosol as detected by immunofluorescence microscopy. This was confirmed by increased cytosolic cathepsin D signal on Immunoblot. Cell viability of cisplatin-treated A549cisR cells was decreased when co-treated with chloroquine, corresponding to a combination index below 0.8, suggesting synergism between the two drugs. Notably, chloroquine activated the mitochondrial cell death pathway as indicated by increase in caspase 9 activity. Interestingly, inhibition of lysosomal proteases using E64 conferred cytoprotection against cisplatin and chloroquine co-treatment, suggesting that chloroquine-induced cell death occurred in a cathepsin-mediated mechanism. Likewise, blockage of caspases partially rescued A549cisR cells against the cytotoxicity of cisplatin and chloroquine combination. Cisplatin promoted a dose-dependent autophagic flux induction preferentially in A549cisR cells, as evidenced by a surge in LC3-II/α-tubulin following pre-treatment with E64 and increase in p62 degradation. Compared to untreated cells, cisplatin induced an increase in cyto-ID-loaded autophagosomes in A549cisR cells that was further amplified by chloroquine, pointing toward autophagic flux activation by cisplatin. Interestingly, this effect was less pronounced in A549Pt cells. Blocking autophagy by ATG5 depletion using siRNA markedly enhances susceptibility to cisplatin in A549cisR cells. Taken together, our results underscore the utility of targeting lysosomal function in overcoming acquired cisplatin refractoriness in lung cancer.

## Introduction

Non-small-cell lung cancer (NSCLC) remains the leading cause of cancer-related deaths in the United States, and claims more lives each year than all other major cancers combined [1]. Metastatic NSCLC harboring EGFR mutation, ALK or ROS1 rearrangement have been successfully treated with tyrosine kinase inhibitors (TKIs) targeting the corresponding molecular aberration [2, 3]. Moreover, checkpoint inhibitors have recently demonstrated promising success in the context of PD-L1 overexpression, or in combination with chemotherapy [4]. However most patients lack these molecular alterations and thus may not respond to these TKIs and/or immunotherapy [2–4]. In such cases, systemic treatment consists mainly of platinum-based therapy [5]. Cisplatin, a drug commonly used in advanced lung cancer therapy, crosslinks purine bases in DNA, resulting in DNA damage, and consequently apoptosis [6]. While approximately 20–40% of patients with advanced NSCLC respond initially to platinum-based chemotherapy, most responses are short-lived accounting for their dismal prognosis [7, 8]. Primary and acquired resistance to cisplatin limit its clinical efficacy. Identification of the biological mechanisms conferring an escape to cisplatin cytotoxicity is a key step to improve treatment efficacy. While reduced cisplatin uptake and increased DNA repair are well-described mechanisms of resistance to cisplatin, emerging evidence shows that enhanced autophagy and defective apoptotic pathways are also implicated [9–11].

There are two major molecular pathways for apoptosis; the extrinsic pathway initiated by activation of death receptors on the cell surface and mediated by caspase 8, and the mitochondrial intrinsic pathway activated by various stimuli such as DNA damage, and involves the release of mitochondrial cytochrome c into the cytosol, leading to formation of the apoptosome and activation of caspase 9 [12, 13]. It is believed that pore formation within the mitochondrial outer membrane, tightly regulated by a balance between various pro-apoptotic (such as Bax and Bak) and anti-apoptotic proteins of the Bcl-2 family, promotes the release of the mitochondrial apoptogenic proteins into the cytosol [14]. Subsequently, the activated caspase 9 or 8 will activate downstream effectors cleavage, leading eventually to activation of caspase 3, the executioner of apoptosis.

Refractory cancer cells often exhibit a reduced expression of pro-apoptotic signaling and/or over-expression of anti-apoptotic proteins, therefore conferring an ability to evade the various apoptotic stimuli of anti-tumor agents [15]. Establishing novel therapeutic strategies to reverse chemo-refractoriness is thus needed.

LMP was proposed as a new strategy to combat cancer drug resistance [16, 17]. Indeed, lysosomes are acidic organelles responsible for the disposal and recycling of damaged macromolecules and organelles and degradation of extracellular materials delivered to them via endocytosis, autophagy or phagocytosis [18]. Its acidic environment contains numerous hydrolases (proteases mainly cathepsins, lipases, and nucleases) responsible for the degradation of various molecules [19]. Lysosomal cell death is initiated by LMP that leads to leakage of lysosomal hydrolases into the cytosol. Whereas massive translocation of lysosomal protease causes cell necrosis, minor leakage can lead to caspase-dependent or caspase-independent apoptosis [20]. An accumulating body of evidence has shown that triggering lysosomal cell death with the usage of molecules that directly disrupt lysosomal membrane can circumvent the already defective apoptotic pathway and re-sensitize multidrug resistant cancer cells to chemotherapy making lysosomotropic agents an attractive strategy in cancer drug discovery [16, 18, 20–22].

Many LMP inducers were shown to induce cell death in different cancer cell types including lung, glioblastoma multiforme and pancreas. As such, curcumin, cardenolide, pterostilbene and microtubule stabilizers are compounds proven to trigger lysosomal cell death in lung cancer [16, 23–25]. The antimalarial medication, chloroquine, was described to synergize with PI3K inhibitors in neuroblastoma cells via lysosomal membrane destabilization [26, 27]. Accumulating evidence revealed that chloroquine can effectively overcome resistance to cisplatin in other malignancies, including gastric, endometrial, urothelial, adrenocortical, melanoma, head/neck and esophageal cancer [28–34]. This effect is reportedly attributed to chloroquine ability to downregulate autophagy by blocking lysosomal fusion with autophagosomes. At present, *the specific role of LMP induction in overcoming acquired resistance to cisplatin in lung cancer has not been established*.

Another mechanism by which cancer cells protect themselves involves autophagy modulation [35]. Autophagy is a catabolic degradation process whereby cellular proteins and damaged organelles are engulfed by autophagosomes, degraded after fusion with lysosomes, and recycled to sustain cellular metabolism. Proteins encoded by autophagy-related genes such as ATG5 and ATG7, regulate this process [36, 37]. Autophagy is considered to have a dichotomous role in cancer depending on cellular context, and the extent of cellular stress [36, 38, 39]. Numerous studies have indicated that autophagy upregulation functions as a protective mechanism that enables cancer cells to adapt under unfavorable conditions such as nutritional deficiency and exposure to chemotherapy and radiation, whereas turning off autophagy facilitates the cytotoxicity of chemotherapy [36, 40–42]. In contrast, other studies have revealed the implication of autophagy activation in cellular degradation and caspase-independent cell death [39, 43]. These studies were confounded by the different methods used to monitor and modulate cellular autophagic activation. *Thus*, *the role of autophagy in cisplatin resistance remains yet to be clarified in lung cancer*.

In this present study, we examined the involvement of defective apoptotic pathways and autophagy upregulation in acquired resistance to cisplatin in lung cancer. In this context, we directly compared the apoptotic and autophagic profiles in response to cisplatin of parental and refractory lung cancer cells. In particular, we provided compelling evidence that chloroquine, through promoting LMP at a larger extent in A549cisR cells, augments the therapeutic activity of cisplatin against these cells via cathepsin-mediated and in part, caspase-independent mechanisms. Collectively, our findings indicate that LMP activation and autophagy inhibition constitute appealing therapeutic modalities to counteract acquired resistance to cisplatin in non-small-cell lung cancer.

## Materials and methods

### Cell culture

The A549Pt and A549cisR non-small-cell lung cancer cell lines were a kind gift from Dr. Martin Barr of the Trinity Translational Medicine Institute, Trinity Center for Health Sciences, St. James Hospital & Trinity College Dublin, Ireland. The A549cisR cell line was established by treating A549Pt cells with repetitive and incremental concentrations of cisplatin as previously described [7]. A549Pt cells were maintained in F-12K media (Corning Life Sciences, Tewksbury, MA) supplemented with 10% fetal bovine serum (FBS), (Gemini Bio-Products, West Sacramento, CA) and 1% penicillin-streptomycin (Cellgro, Manassas, VA). For A549cisR cells, the F-12K media was supplemented with 4mM glutamine (Cellgro, Manassas, VA) and 5.95 μM cisplatin. Cells were cultured in a humidified atmosphere of 5% CO_2_ at 37°C.

### Chemicals, reagents and antibodies

Chloroquine (CQ), staurosporine (STP), E64 and cisplatin (cis-diaminedichloroplatinum (II), cisPt) were obtained from Sigma-Aldrich (St. Louis, MO). Dextran fluorescein 40,000 MW and DAPI (4’, 6-diamidino-2-phenylindole) were purchased from Life Technologies (Grand Island, NY). The pan-caspase inhibitor z-VAD-FMK was purchased from ApexBio Technology (Houston, TX). The primary antibodies used in this study includes the following: caspase 3 (3G2), LC3 A/B, ATG5 (D5F5U) and p53 (1C12) from Cell Signaling Technology (Beverly, MA), cathepsin D (H-75) and Bax (N-20) from Santa Cruz Biotechnology (Santa Cruz, CA), LAMP-1 (H4A3) from Developmental Studies Hybridoma Bank at the University of Iowa, BAK from EMD Millipore (Billerica, MA), and α-Tubulin Ab2 (clone DM1A) from Fisher Scientific (Rockford, IL). The secondary antibodies, horseradish peroxidase conjugated anti-rabbit and anti-mouse, were both from GE Healthcare (Pittsburgh, PA). ECL2 was purchased from Thermo Fisher Scientific (Rockford, IL).

### Cell proliferation assay

Measurement of cell viability was determined using the CellTiter-Blue^®^ assay. Briefly, A549Pt and A549cisR cells were plated in 96-well plates and incubated overnight at 5% CO_2_ at 37°C. Next day, cells were treated with different treatment conditions as described in the “results” section. Following various periods of time, CellTiter-Blue^®^ (Promega, Madison, WI) was added to each well, according to the manufacturer’s instructions. The plate was incubated at 37°C for 1 hour, and the fluorescence was measured using a Synergy 4 fluorescence plate reader. Combination index was calculated as a ratio of the actual viability to the expected viability. The expected survival rate was estimated using the fractional product method [44].

### Western blot analysis

Western blot analysis was conducted as previously described [45]. Briefly, A549Pt and A549cisR cells were plated to approximately 70% confluency. Next day, cells were exposed to the designated treatment as described in the “Results” section. At the end of the incubation time, cells were lysed in 4X Laemmli buffer (0.125 M tris-HCl, pH 6.8, 4% SDS, 0.13mM bromophenol blue, 1 M sucrose) containing 0.5% ß-mercaptoethanol and boiled for approximately 5 minutes. Whole protein lysates were separated on a 10% polyacrylamide gel by SDS-PAGE, transferred onto PVDF membranes (Millipore, Billerica, MA), then blocked for 1 hour in 10% milk in TBST (20 mM Tris, 137 mM NaCl, 0.1% Tween 20, pH 7.5) and probed with appropriate primary antibodies for 16 hours. Membranes were then incubated with HRP-conjugated secondary antibodies (1:5000) for one hour prior to signal development using ECL2. Kodak BioMax XAR film was used for chemiluminescence detection.

### Detection of lysosomal membrane permeabilization

Visualization of LMP was undertaken with a method modified from Jäättelä and Nylandsted [46]. Briefly, A549Pt or A549cisR cells were grown overnight in 96-well plates. Next day, cells were loaded with dextran fluorescein 40 KDa (FITC dextran) for 16 hours. Following a 6 hours chase period, a designated treatment was applied. At the end of experimentation, cells were fixed in ice-cold PBS containing 4% paraformaldehyde for 10 minutes. Cells were then washed with PBS and incubated with Hoechst (2μg/ml) for 30 minutes to stain the nuclei. Plates were mounted on a Cellomics ArrayScan automated fluorescence imager (Thermo Fisher Scientific, Inc., Waltham, MA). Cells were photographed using a 40× objective in 2 fluorescent channels and images of a total of 20 different fields per well were captured. Increased green fluorescence throughout the cytoplasm and loss of the punctae pattern indicate leakage of FITC dextran from the lysosomes, a surrogate of LMP.

### Cytosolic and lysosomal proteins fractionation

Cells were plated in P100 dishes and grown to 90% confluency. Next, cells were treated with the appropriate drugs as described in the “Results” section. After overnight incubation, cells were scraped in cold 1x PBS and collected by centrifugation at 1600rpm. The cell pellet was resuspended in homogenization buffer (20mM Hepes, 0.25M sucrose, 1mM EDTA, pH 7.4) containing protease inhibitor cocktail (Roche, Diagnostic Corporation, Indianapolis, IN) and cells were lysed with 80 passages through a 23G needle. The homogenate was centrifuged at 2700rpm for 10 minutes to pellet the unbroken cells and the nuclei. The supernatant was transferred to Beckman Coulter centrifuge tubes and the cytosolic and lysosomal fractions were separated by ultracentrifugation at 100,000g for 1 hour. The supernatant representing the cytosolic fraction and the pellet representing the lysosomal fraction were collected, and combined with the 4x Laemmli buffer and analyzed by Western Blot for cathepsin D, LAMP-1 (lysosomes) and α-tubulin (cytosolic marker).

### Apoptosis assays

A549Pt and A549cisR cells were plated in 6-well plates and treated for 48 hours with the designated drugs as described in the “results” section. Samples of treated cells were prepared according to the manufacturer’s instructions. Briefly, cells were collected by trypsinization, and pelleted by centrifugation for 5 minutes at 2000 rpm, suspended and mixed in Annexin-V binding buffer for a final concentration of 2x10^6^ cells/ml. Samples were then incubated at room temperature for 15 minutes with Annexin V-FITC and Propidium iodide (PI) solution. After 15 minutes of incubation at room temperature and in the dark, cells were washed with 1x PBS, pelleted by centrifugation and re-suspended in Annexin V buffer prior to the measurement of fluorescence. In parallel, cell suspensions were incubated for 1 hour with FITC-FMK-specific substrates to detect the active form of caspase 3, 8 or 9, according to the manufacturer’s instructions (CaspGLOW^™^ Fluorescein Active Caspase-3, 8 or 9 staining kits from BioVision Incorporated, Milpitas, CA). Cell suspensions were immediately loaded into the counting chamber of the Vision-CBA Cellometer (Nexcelom Bioscience, Lawrence, MA), an automated imaging-based fluorescence microscope equipped with bio-analysis cell counter system, and analyzed by FCS express 4 analysis software, as described by the manufacturer.

### Autophagy assay

To measure the level of autophagosome formation, cells were stained with Cyto-ID green autophagy dye (Enzo Life Sciences, Farmingdale, NY), a cationic amphiphilic tracer that diffuses into autophagosomes, as per manufacturer’s instructions. Briefly, the cyto-ID green solution was prepared by mixing the dye with 1x assay buffer. Following trypsinization, the sample of treated cells was centrifuged, and the dye added. The sample was incubated for 30 minutes at 37°C, in the dark, followed by a wash and re-suspension in 1x assay buffer. The cell suspension was immediately loaded into the counting chamber of the vision-CBA Cellometer, a platform that detects the CytoID fluorescence signals from each counted cell, and generates fluorescence histograms comparable to flow cytometry. The autophagic flux was evaluated by immunoblotting cell lysates for LC3-II before and after exposure to E64, a known lysosomal protease inhibitor, and evaluating the rate of increase in LC3-II signal [47]. The autophagic degradation was assessed by immunoblotting for p62 protein.

### ATG5 silencing

A549cisR cells were plated in 96-well plates and were treated the following day with 25 μM cisplatin. After 24 hours, media was removed and cells were transfected with 50nM ON-TARGET*plus* SMARTpool ATG5 or non-targeting (NT) siRNA according to the manufacturer’s instructions (GE Dharmacon, Lafayette, CO). Cells were incubated for 48 hours with the siRNA, media was changed to 1% FBS media containing various concentrations of cisplatin, and cells were incubated for an additional 48 hours with the drug. At the end of experimentation, cell viability was measured using the CellTiter blue assay.

### Lysosomal number estimation

The number of lysosomes was quantified by the Cellomics imager as previously described [45]. Briefly, cells were grown on 96-well plates. Following fixation, nuclei and lysosomes were stained with DAPI and LAMP-1 antibodies, respectively. Plate was mounted on the Cellomics platform. Biocompartmental analysis calculated the number of lysosomes inside a virtual ring of 12 pixels width, and of inner rim 6 pixels away from the nucleus, representing the number of lysosomes inside the cytosol.

### Statistical methods

All statistical analyzes were performed using GraphPad Prism 7.0 and Microsoft Excel software. One or two-tailed Student’s t-tests were used to analyze statistical differences. The independent influence of ATG5 depletion on cisplatin-treated cell viability was assessed by two-way ANOVA. This was followed by Sidak’s multiple comparisons test to compare cell viability between ATG5 and NT siRNA-transfected cells treated with the same drug concentration. 1-way ANOVA test was utilized to statistically analyze the difference in viability among ATG5 or NT siRNA-transfected cells treated with various concentration of cisplatin. All quantitative data are shown as Mean ± Standard error of the mean (SEM) from three independent experiments. All statistically significant differences are annotated within the figures. Differences at p<0.05 were considered statistically significant. The half-maximal inhibitory concentration (IC_50_) of the drugs was calculated on GraphPad Prism using the log(inhibitor) vs. response—variable slope (four parameters) equation under the nonlinear regression dialogue.

## Results

### Resistance to cisplatin correlates with defective apoptotic response

To determine their IC_50_ values, we initially treated A549Pt and A549cisR cells with increasing concentrations of cisplatin ranging from 0.1 to 1000 μM. Cell viability was estimated by CellTiter-Blue^®^ assay at 24, 48 and 72 hours. The IC_50_ concentration of cisplatin in A549cisR cells was 47 μM compared to 13 μM in parental cells (S1 Fig).

**Fig 1:**
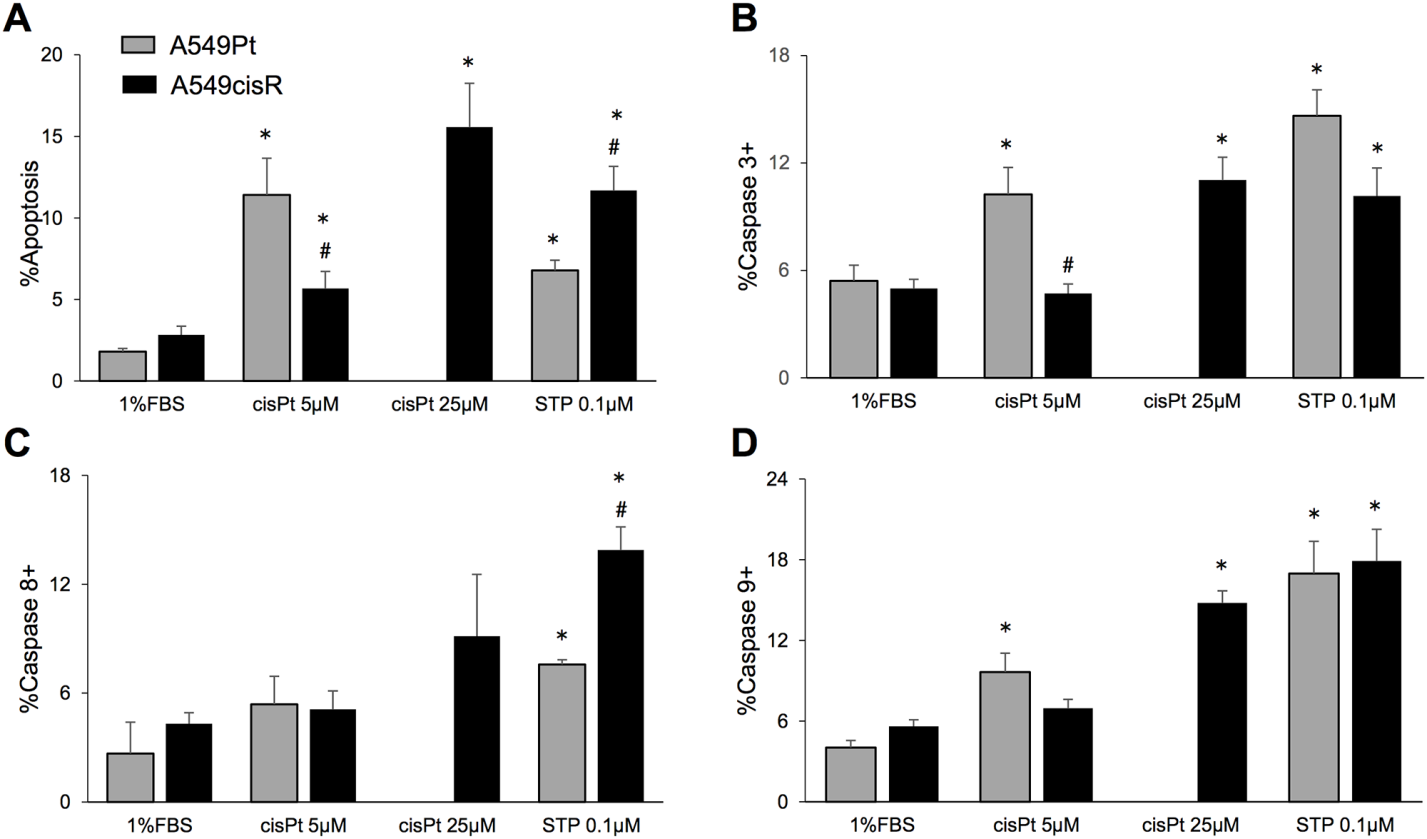
A549cisR cells exhibit defective apoptosis following exposure to cisplatin. A549Pt and A549cisR cells were treated for 48 hours with 5 μM and 5 μM or 25 μM cisplatin (cisPt), respectively. Cells cultured in 1%FBS were used as negative control, and cells treated with staurosporine (STP, 0.1 μM) were used as positive control. Subsequently, cell suspensions were incubated with Annexin V-FITC/PI or FITC-FMK-specific substrates for the detection of apoptosis or active caspases 3,8 and 9, respectively. The percentage of cells with increased Annexin-V/low PI staining (A), and increased caspases activity (B-D) measured by the Cellometer platform are plotted. Graph bars represent the Mean±SEM from at least three independent experiments. *p<0.05 treatment vs. 1%FBS, ^#^ p<0.05 A549Pt vs. A549cisR for the same treatment conditions.

One of the hallmarks of refractory cancer cells is aberrant apoptosis in response to chemotherapy. We thus sought to interrogate the apoptotic response to cisplatin after 48 hours treatment with 5 μM in A549Pt cells, 5 μM or 25 μM in A549cisR cells (Fig 1). 1%FBS was used as negative control, and staurosporine (0.1 μM) was used as positive control. By measuring the percentage of Annexin-V stained cells, cisplatin-induced apoptosis was increased in both cell lines, however we observed a higher percentage of Annexin-V stained cells in A549Pt cells treated with 5 μM of cisplatin when compared to A549cisR cells treated with the same concentration of drug (Fig 1A). Similarly, cisplatin-induced caspase 3 and 9 activation was effectively increased in A549Pt cells upon treatment with 5 μM cisplatin, as measured with Cellometer (Fig 1B, 1C and 1D). While A549cisR displayed a significant increase in caspase 3 and 9 activity when treated with 25 μM cisplatin, this activation was trivial in response to 5 μM cisplatin. No significant increase in caspase 8 activity was observed in A549Pt and A549cisR cells (Fig 1C). Taken together, these data indicate a defect in the intrinsic apoptotic response in A549cisR cells.

Since a previous study showed that cisplatin-induced apoptosis in parental A549 cells occurs via p53-dependent Bak and p53-independent Bax activation [48], we evaluated whether p53, Bak and Bax activation following cisplatin treatment are altered in A549cisR cells. We therefore analyzed and compared the expression of p53, Bak and Bax proteins in response to 10 μM cisplatin treatment in both A549cisR and A549Pt cells at different time points. We noticed a gradual and clear increase in p53 protein expression across different time points in A549cisR cells. In particular, this increment in p53 expression was greater in resistant cells when compared to their parental counterparts. In contrast, the induction of Bax and Bax expression was greater in A549Pt cells in response to cisplatin treatment (S2 Fig).

### Chloroquine induces preferential lysosomal membrane permeabilization and cytosolic translocation of cathepsin D in A549cisR cells

**Fig 2:**
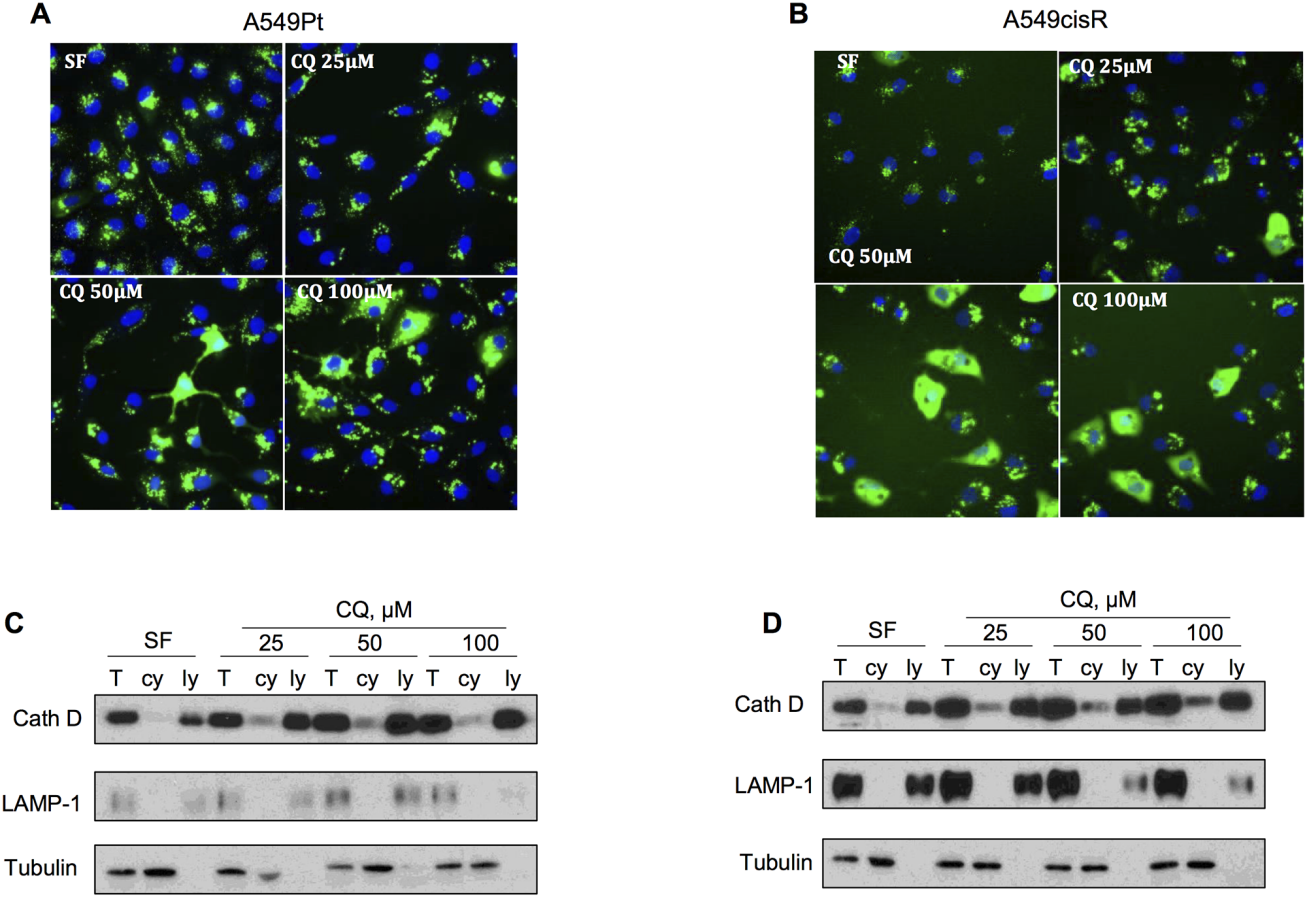
Chloroquine triggers lysosomal membrane permeabilization in A549cisR cells, promoting the release of lysosomal cathepsins into the cytosol. A549Pt **(A)** and A549cisR **(B)** cells were loaded with FITC-dextran 40 kDa for 16 hours, and treated overnight with different concentrations of chloroquine (CQ) in serum-free media. Cells were then fixed, nuclei were stained with Hoechst, and plate was mounted on Cellomics ArrayScan automated fluorescence imager. Representative immunofluorescence microscopy images are shown. **(C-D)** A549Pt (C) and A549cisR (D) cells were treated overnight with different concentrations of chloroquine in serum-free media. Next day, cytosolic (cy) and lysosomal (ly) or total protein (T) fractions were separated by ultracentrifugation and lysates were loaded on SDS/PAGE gels. Upon transfer, membranes were probed with specific antibodies against cathepsin D, LAMP-1 (lysosomes) and α-tubulin (cytosolic marker).

Previous data have shown the ability of chloroquine to disrupt lysosomal membrane by its detergent-like property producing lysosomal protease translocation into the cytosol [26]. While it was previously suggested that LMP inducers preferentially target lysosomes of cancer cells compared to normal cells [49], whether lysosomes derived from A549cisR cells are more vulnerable to chloroquine, is currently unknown. We therefore compared the ability of chloroquine to trigger LMP in both A549Pt and A549cisR cells. To monitor for LMP, lysosomes were labeled with FITC-dextran, and cells were treated overnight with different concentrations of chloroquine. Fluorescent images acquired by Cellomics revealed a dose-dependent increase in cytosolic green hazy fluorescence and loss of the punctae pattern of the dextran-loaded lysosomes, reflecting the leakage of dextran from lysosomes into cytosol as a result of LMP (Fig 2A and 2B). Interestingly, this effect was clearly more pronounced in A549cisR cells (Fig 2B). In order to further confirm the occurrence of cathepsin D translocation as a direct consequence of lysosomal membrane damage, cytosolic extraction by cellular sub-fractionation was immunoblotted for cathepsin D, LAMP-1 and α-tubulin. As demonstrated in Fig 2D, the addition of chloroquine markedly increased cytosolic cathepsin D signal in A549cisR, in a dose-dependent fashion. Interestingly, this effect was less prominent in A549Pt cells (Fig 2C). The lack of cytosolic contamination by lysosomes as a result of poor separation was confirmed by the absence of LAMP-1 signal in the cytosolic fraction. Collectively, our results confirmed the ability of chloroquine to preferentially trigger LMP in A549cisR cells and to a lesser extent in A549Pt cells.

### A549cisR cells exhibit an increase in lysosomal number compared to parental cells

**Fig 3:**
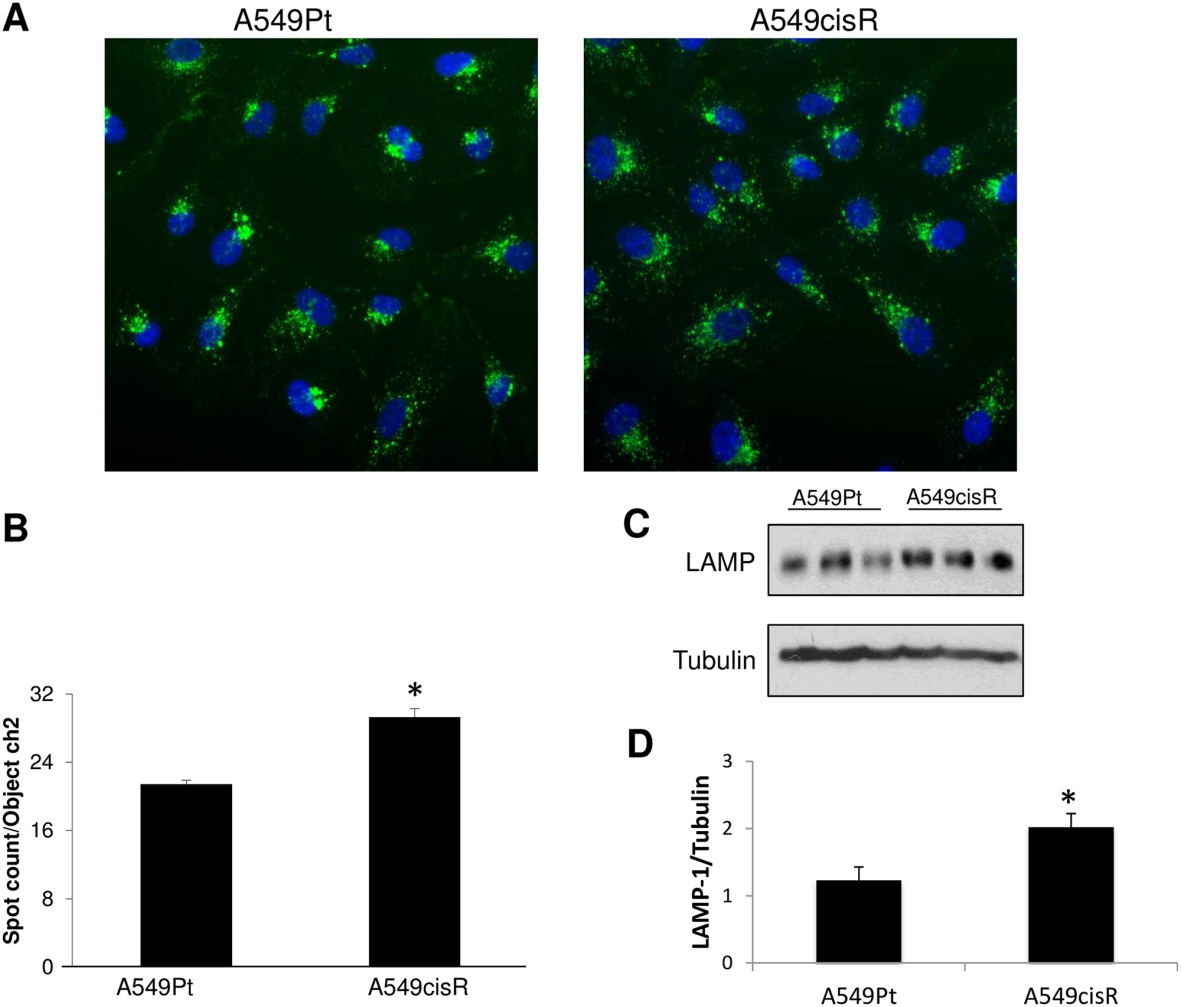
A549cisR cells exhibit an increase in the number of lysosomes. **(A)** Untreated A549Pt and A549cisR cells were stained for DAPI (blue) and LAMP-1 (green), and immunofluorescent images were captured using Cellomics (40x objective) in two fluorescent channels. **(B)** The number of lysosomes was quantified by the Cellomics imager using the biocompartmental analysis algorithm. **(C)** Cells lysates from untreated A549Pt and A549cisR cells were collected and Western blot analysis was performed for LAMP-1 and α-tubulin. **(D)** Graph displaying LAMP-1 level adjusted to tubulin level as quantified by WB densitometry. Graph bars represent the Mean±SEM from three independent experiments. *p<0.05 A549Pt vs. A549cisR.

We next aimed to examine whether the augmented susceptibility of A549cisR cells to chloroquine-induced LMP is due to an increase in lysosomal mass. We therefore utilized the imaging-based computerized platform, Cellomics, to measure the number of cytosolic lysosomes in both untreated A549Pt and A549cisR cells. A549cisR displayed markedly greater lysosomal numbers, and this can be readily seen in Fig 3A and 3B. To further confirm this observation, whole cell lysates from untreated cells were immunoblotted for LAMP-1, a specific marker of lysosomal membrane. A549cisR cells exhibited a significantly higher LAMP-1 signal in comparison to parental cells, which may be due to an increase in lysosomal mass in resistant cells (Fig 3C and 3D). It is conceivable that the higher number of lysosomes in A549cisR cells has likely resulted into their distinct vulnerability to LMP, although an impairment affecting the various factors involved in lysosomal membrane integrity could not be ruled out. Taken together, these results suggest a possible association between the number of lysosomes and the magnitude of chloroquine-induced LMP.

### Chloroquine sensitizes A549cisR cells to cisplatin in an LMP-mediated manner

**Fig 4:**
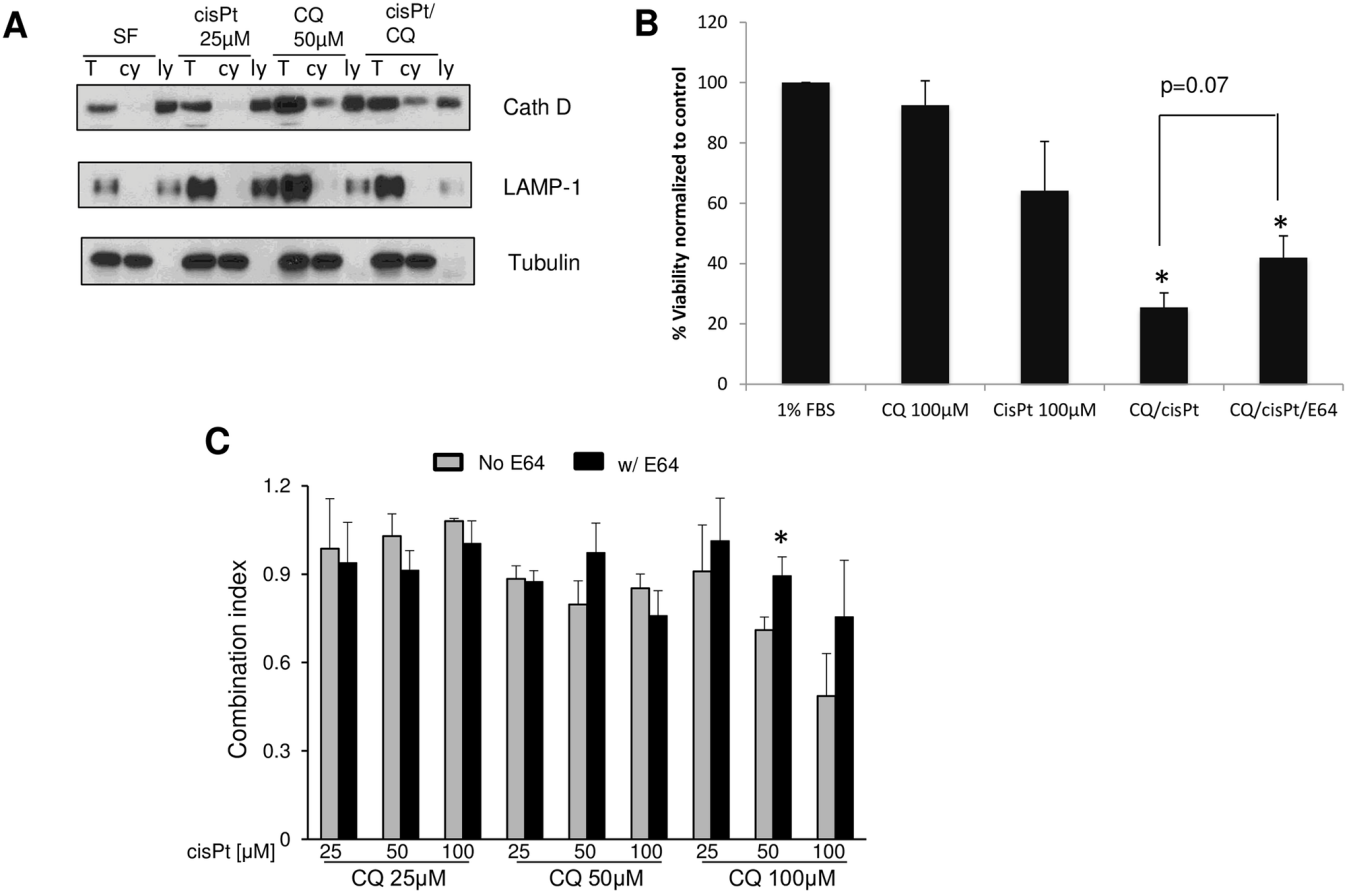
Chloroquine potentiates cisplatin cytotoxicity against A549cisR lung cancer cells in an LMP-mediated manner. **(A)** A549cisR cells treated overnight with cisplatin and chloroquine. Next day, cytosolic (cy), total protein (T) and lysosomal (ly) fractions were separated by ultracentrifugation and lysates were loaded on SDS/PAGE gels. Upon transfer, membranes were probed with specific antibodies against cathepsin D (cath D), LAMP-1 (lysosomes) and α-tubulin (cytosolic marker). **(B)** A549cisR cells were treated with 1%FBS, chloroquine 100 μM, cisplatin 100 μM, or combination with or without pretreatment with E64 (10 μM). Cell viability was measured with CellTiter-Blue^®^. X-axis, represent the % cell viability at 48 hours (T48/T1) normalized to the negative control 1%FBS. *p<0.05 treatment condition versus 1%FBS. **(C)** A549cisR cells were treated with different concentrations of cisplatin (cisPt) and CQ and pre-treated or not with E64 (10 μM) for 2 hours. Combination index (CI) was calculated as the ratio of the actual cell viability to the expected cell viability. Graph bars show the Mean±SEM from three independent experiments. *p<0.05 cells pre-exposed to E64 versus cells not pre-exposed to E64 and treated with the same concentration of cisplatin and chloroquine.

Based on the differential impact of chloroquine on degree of LMP between parental and resistant cells, the ability of chloroquine to restore sensitivity to cisplatin in lung cancer cells was examined. We therefore treated A549cisR cells with different concentrations of chloroquine, cisplatin or a mix of the two drugs and evaluated their effects on cell viability. Chloroquine at a dose of 100 μM exerted slight cytotoxicity when used alone (Fig 4B). However, when combined with cisplatin, it reduced A549cisR cell viability relatively to cells treated with cisplatin alone. Interestingly, this potentiating effect of chloroquine was more effective at higher concentrations of cisplatin (Fig 4B and 4C). For instance, the combination index of cisplatin 50 μM (concentration close to its IC_50_) and chloroquine 100 μM at 48hours was 0.71, indicating synergism between the 2 drugs. In particular, the combination index for cisplatin 100 μM and chloroquine 100 μM at 48 hours was 0.48, highlighting a significant synergism between the two drugs (Fig 4C). To confirm the occurrence of LMP with the drugs mix, cathepsin D was measured in the cytosolic extract of cells treated with chloroquine or cisplatin, alone or combined. As expected, a translocation of cathepsin D into the cytosol was observed in cells treated with either chloroquine or chloroquine/cisplatin combination, but not in cells treated with cisplatin alone (Fig 4A). Chloroquine has been previously reported to act in concert with chemotherapy to induce cell death. However, most of the potentiating effects of chloroquine are primarily attributed to its ability to downregulate autophagy [28–34]. To further determine the contribution of leaked cathepsin in mediating the cooperation between chloroquine and cisplatin, we pre-treated A549cisR cells with 10 μM of the lysosomal proteases neutralizer E64 for 2 hours prior to treatment with cisPt and/or CQ, and monitored its effect on cell death. Interestingly, E64 conferred cytoprotection for cells co-treated with cisplatin and chloroquine (Fig 4B). This was manifested by an attenuation of cell death and increase in combination index value in the presence of E64 (Fig 4B and 4C). Interestingly, synergism between chloroquine and cisplatin was minor in A549Pt (data not shown), and this may be due to the attenuated level of LMP seen in these cells. Taken together, our results clearly demonstrate that chloroquine-induced cell death occurs in a cathepsin-mediated manner, as a result of chloroquine-triggered lysosomal membrane disruption.

### The cytotoxic effect of chloroquine and cisplatin combination is partially dependent on caspase activation

**Fig 5:**
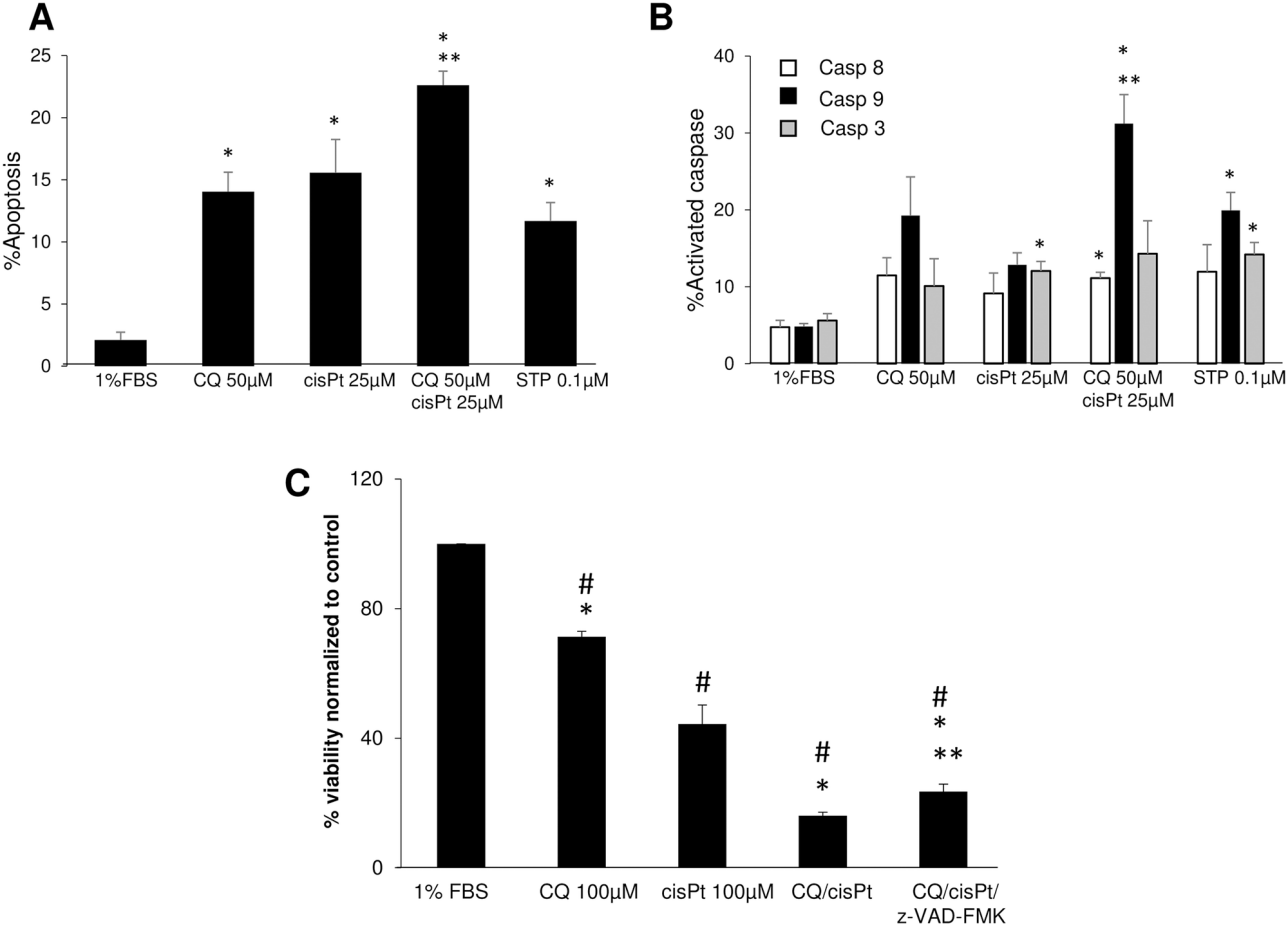
Treatment with pan-caspase inhibitor partially rescues A549cisR cells against cisplatin and chloroquine. **(A, B)** A549cisR cells were treated for 48h with chloroquine (CQ, 50 μM), cisplatin (cisPt, 25 μM) or the drug combination. Staurosporine, STP, (0.1 μM) represents the positive control. Subsequently, cell suspension was incubated with Annexin V-FITC/PI or FITC-FMK-specific substrates for apoptosis **(A**) and caspase **(B)** assays, respectively. Next, samples from each condition were mounted on the Cellometer imager to measure the fluorescence of which the values are plotted in graphs **A** and **B**. Graph bars represent the Mean±SEM from three independent experiments. *p<0.05 treatment vs. 1%FBS. **p<0.05 chloroquine/cisplatin vs. cisplatin only. **(C)** A549cisR cells were treated with chloroquine 100 μM, cisplatin 100 μM, pretreated or not with the pan-caspase inhibitor Z-VAD-FMK. Cell viability was measured with CellTiter-Blue^®^. The X-axis represents the percentage of cell viability at 48 hours (T48/T1) normalized to the negative control 1%FBS. Quantitative data are reported as Mean±SEM from three independent experiments. ^#^p<0.05 1%FBS versus treatment, *p<0.05 cisplatin versus other treatments, **p<0.05 chloroquine/cisplatin versus chloroquine/cisplatin/Z-VAD-FMK.

We have so far observed a defect in the activation of apoptotic pathways in A549cisR cells treated with cisplatin. We next sought to elucidate whether the addition of chloroquine to cisplatin can induce apoptosis in A549cisR cells. In doing so, we measured Annexin-V/PI, and the activation of caspase 3, 8 and 9 using the two drugs separately or in combination. Although the synergistic effect of cisplatin and chloroquine was observed only at CQ dose of 100 μM, we choose a lower concentration of 50 μM that is less toxic to the cells so we can have more consistent measurement on caspases activities. Notably, we found a greater number of Annexin-V stained/low PI apoptotic cells (Fig 5A) accompanied by a corresponding greater activation of caspase 9 in A549cisR cells when co-treated with both drugs, compared to cells treated with cisplatin only, and untreated cells (Fig 5B). We also noticed a trend towards an increased caspase 3 activity, but the results variability may have explained the lack of statistical significance. Collectively, our findings indicate that chloroquine exacerbates the activation of the intrinsic apoptotic pathway when used in combination with cisplatin.

Previous reports have demonstrated that lysosomal membrane permeabilization can trigger caspase-dependent and caspase-independent cell death [20]. We also observed an activation of caspase 9 in cells co-treated with cisplatin and chloroquine (Fig 5B). To determine if caspases are in fact necessary for chloroquine-induced cell death in the presence of cisplatin, we pretreated cells for 1 hour with the pan-caspase inhibitor, z-VAD-FMK, prior to incubation with cisplatin and chloroquine. As expected, treatment with cisplatin and chloroquine reduced cell viability compared to each drug alone. Interestingly, pre-treatment with caspase inhibitors partially rescued A549cisR cells, as shown in Fig 5C. As a matter of fact, viability of cells treated with CQ/CisPt/z-VAD-FMK remained significantly inferior to cells treated with cisplatin alone, or untreated cells.

### A549cisR cells exhibit a pronounced increase in autophagic flux in response to cisplatin

Since chloroquine is also known as an autophagy inhibitor [50], we next examined if autophagy plays a role in acquired resistance to cisplatin. For that purpose, we monitored the autophagic activity at baseline and following cisplatin exposure in both A549Pt and A549cisR cells, in serum free and 1%FBS media as well. Autophagic flux is commonly assayed by determining the kinetics of LC3-II protein expression before and after blockade of lysosomal degradation. The microtubule associated protein 1 light chain 3 (LC3) is an autophagy related protein. During autophagosomal formation, the cytosolic form of LC3 (LC3-I) is cleaved and conjugated to phosphatidylethanolamine to form LC3-II, which in turn, is recruited to the autophagosomal membrane. LC3-II remains on autophagosome until its degradation following fusion with lysosomes. Thus, LC3-II is an indicative of autophagosomal formation. Blockade of lysosomal degradation causes accumulation of LC3-II, which will be further amplified upon activation of the autophagic flux [47, 50, 51].

**Fig 6:**
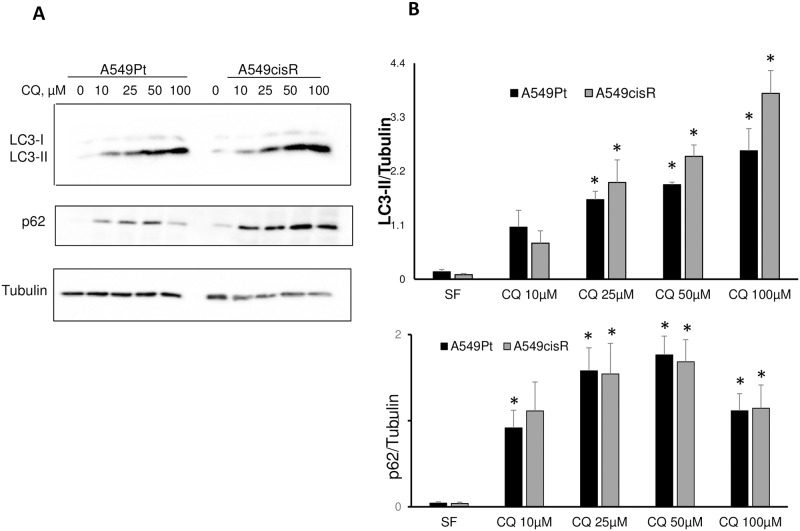
Chloroquine blocks autophagy in A549cisR and A549Pt cells. **(A)** A549Pt and A549cisR cells were plated in 24-well plates and grown for 24 hours, then treated overnight with different chloroquine concentrations. Next, whole cell lysates were prepared and Western blot analysis was performed for LC3-II, p62 and α-tubulin. **(B)** Protein level was quantified by Western blot densitometry. Graph bars represent Mean±SEM from three independent experiments. *p<0.05 SF versus treatment.

We examined the effect of different concentrations of chloroquine on autophagy, by measuring LC3 conversion and p62 level, indicators of autophagosomal accumulation and autophagic degradation respectively. As expected, we observed a marked increase in p62 and noticeable dose-dependent increase in LC3-II when A549Pt and A549cisR in serum free condition were exposed overnight to chloroquine at a concentration as low as 10 μM, clearly indicating a blockade of autophagic flux. Interestingly, the ratio LC3-II/tubulin was higher in chloroquine-treated A549cisR compared to parental A549 cells at 50 and 100 μM, with a trend towards statistical significance (p-value of 0.06 and 0.07 respectively). This observation could be explained by a higher ability of resistant cells to trigger the autophagic flux upon starvation, leading to a higher autophagosomal accumulation following blockage of autophagy with chloroquine (Fig 6).

**Fig 7:**
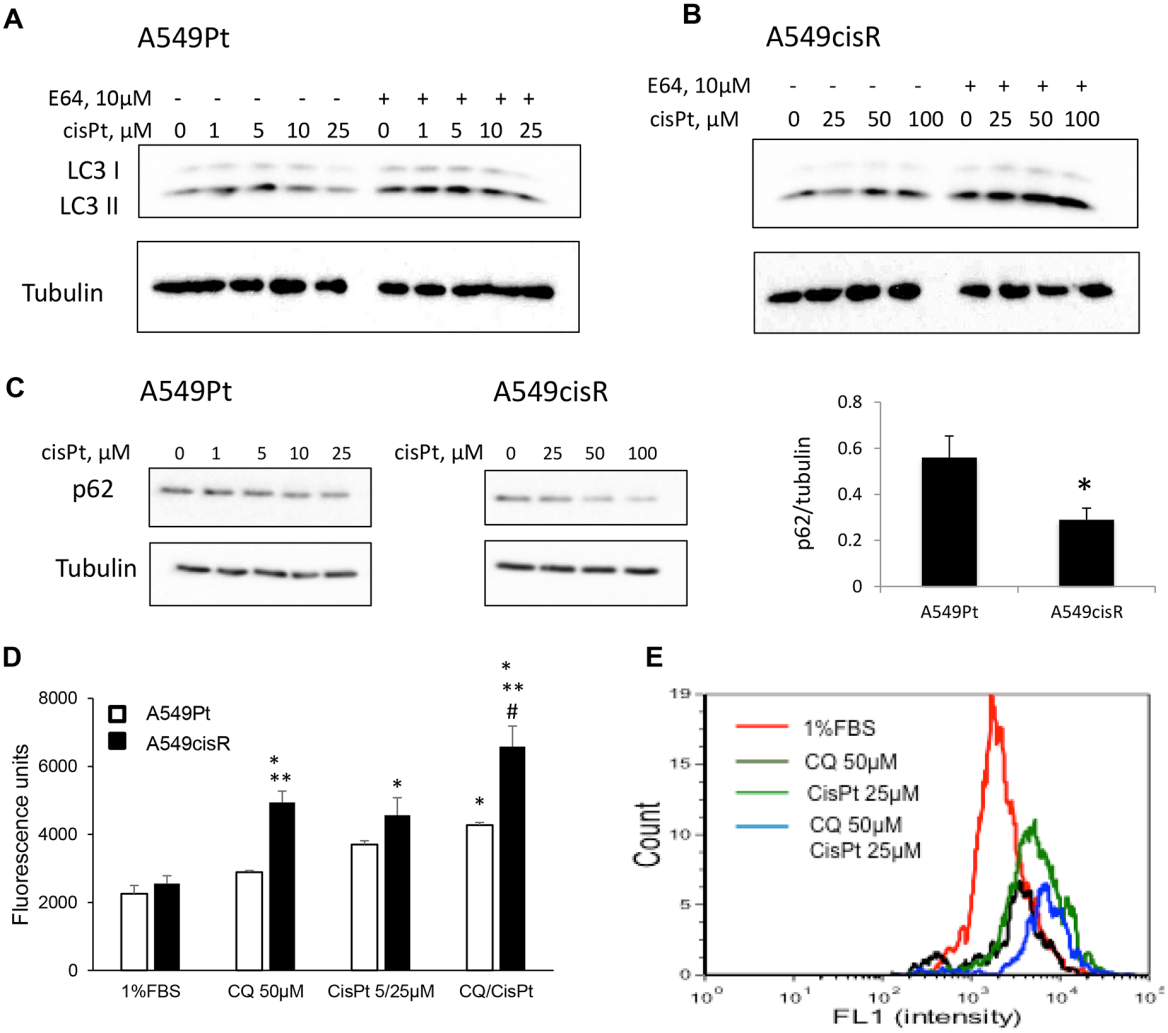
A549cisR cells exhibit increased autophagic flux following exposure to cisplatin, an effect that is less pronounced in parental cells. **(A, B)** A549Pt and A549cisR cells were plated in 24-well plates and grown for 24 hours, then treated overnight with different cisplatin concentrations in 1% FBS media; whenever present, E64 (10 μM) was added for 2h prior to the cisplatin treatment. Next, whole cell lysates were prepared and Western blot analysis was performed for the indicated proteins. **(C)** Whole cell lysates of cells treated with different concentration of cisplatin without E64 were extracted and western blot was performed for p62 and α-tubulin. Levels of p62 and α-tubulin were determined by densitometry. The graph on the right panel represents the ratio of p62/α-tubulin normalized to the negative control 1% FBS in A549Pt and A549cisR cells exposed to cisplatin at concentration close to their corresponding IC_50_ (10 μM and 50 μM respectively). Quantitative data are reported as Mean±SEM from three independent experiments. *p<0.05 A549Pt versus A549cisR. (**D)** A549Pt and A549cisR cells were treated for 48 hours with CQ (50 μM), cisplatin (5 μM for A549Pt or 25 μM for A549cisR) or a drug combination. 1%FBS was used as a negative control. Following incubation with Cyto-ID green detection reagent, cell suspensions were mounted on a Cellometer imager and fluorescence was measured. Y-value depicts the fluorescence value of cyto-ID that stains autophagosomes. Graph bars represent Mean±SEM from three independent experiments. *p<0.05 1%FBS vs. treatment, **p<0.05 A549Pt vs. A549cisR and ^#^p<0.05 chloroquine/cisplatin combination vs. cisplatin alone. **(E)** The histogram plot generated by the Cellometer in A549cisR cells following incubation with different treatment conditions.

We next interrogated the differential effect of cisplatin on autophagic response in both cell lines. We observed a dose-dependent increase in autophagosomal formation in both A549Pt and A549cisR cells upon exposure to different concentration of cisplatin in 1%FBS media. This was demonstrated by a significant increase in the LC3-II/α-tubulin ratio following E64 pre-treatment, an effect that appeared more pronounced in A549cisR cells (Fig 7A and 7B). Similarly, upon exposure to different concentrations of cisplatin, A549cisR cells exhibited a greater reduction in p62/α-tubulin level in comparison to parental cells, implicating a superior activation of autophagic degradation (Fig 7C). Similar experiments conducted in serum free media, a starvation condition that mimics the intra-tumoral microenvironment, revealed a preferential stimulation of autophagic flux in resistant cells in the presence or not of cisplatin, as marked by a higher increment in LC3-II level following E64 pre-treatment (S3 Fig). Next, a fluorescence-based approach was used to study the autophagic flux in both cell lines. Compared to untreated cells, cisplatin induced an increase in autophagosome formation in A549cisR cells, as evidenced by an enhanced cytoID signal. To examine whether this effect was attributable to autophagic flux stimulation or autophagosome accumulation secondary to autophagic degradation blockade, cells were co-treated with cisplatin and chloroquine. This resulted in a marked increase in the cyto-ID signal in comparison to each drug alone, suggesting that cisplatin promoted autophagic flux activation in A549cisR cells. Interestingly, the increased Cyto-ID signal induced by cisplatin in combination with chloroquine, or alone, was inferior and less dramatic in A549Pt cells (Fig 7D and 7E). Likewise, treatment with chloroquine only promoted a higher level of cytoID signal in A549cisR when compared to A549Pt cells in 1%FBS media (Fig 7D), suggesting that baseline autophagic flux is more elevated in resistant cells.

### Suppression of autophagy through ATG5 silencing potentiates cisplatin-mediated cell death in A549cisR cells

**Fig 8:**
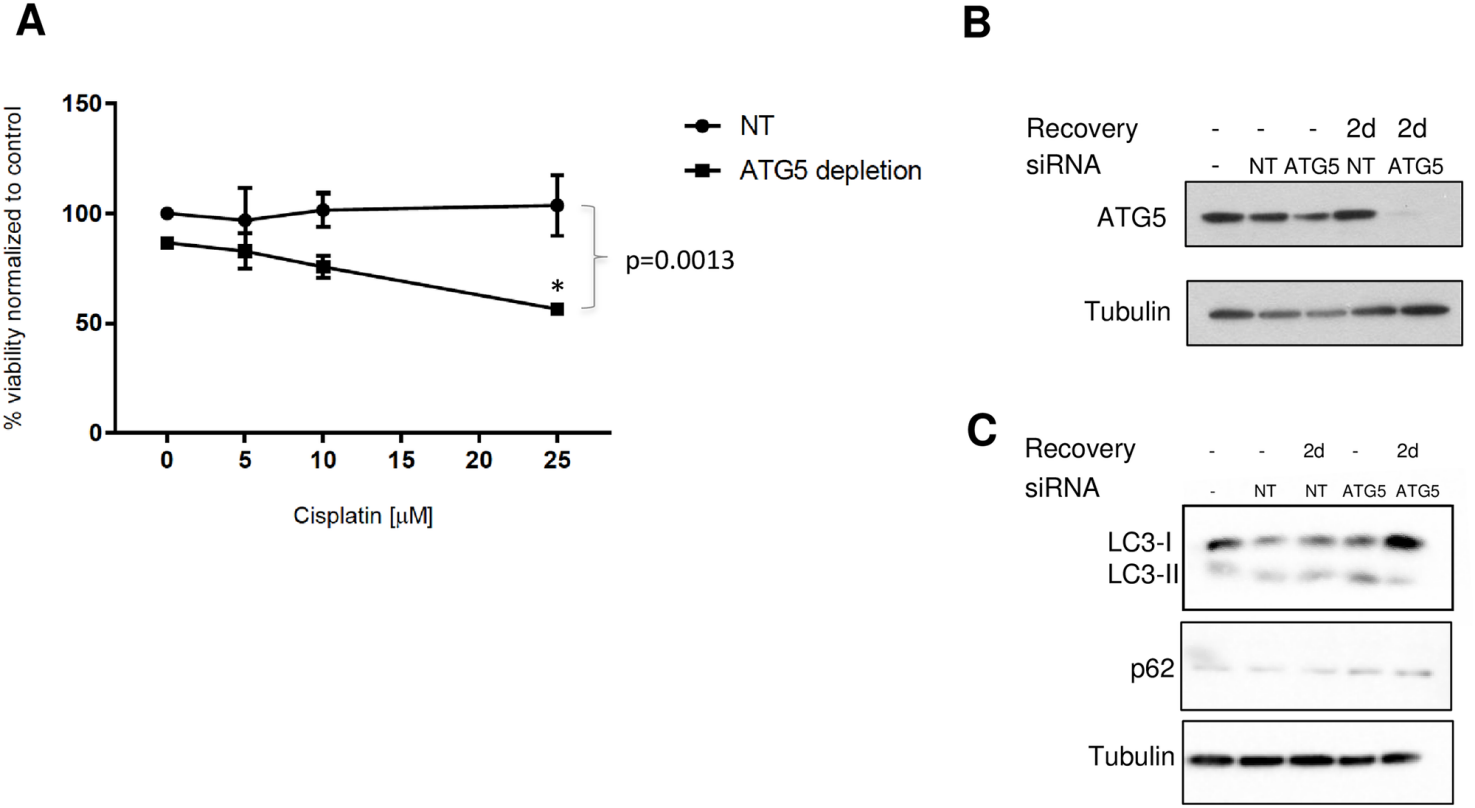
Inhibition of autophagy using ATG5 siRNA potentiates the activity of cisplatin against A549cisR cells. **(A)** A549cisR cells were treated for 24 hours with 25 μM cisplatin prior to transfection with ATG5 siRNA or NT siRNA control. Following two days incubation, media was removed and cells were treated with different cisplatin concentrations for an additional 24 or 48 hours. At the end of the incubation, cell viability was measured using CellTiter-Blue^®^ reagent. Experiments were performed in 1%FBS media. The X-axis represents the percentage of cell viability at 24 hours (T24/T1) normalized to the negative control 1%FBS. Error bars represent SEM from three independent experiment. p-value corresponding to the difference between ATG5 and NT siRNA-transfected cells was obtained by 2-way ANOVA. p-value corresponding to the individual difference between ATG5 and NT siRNA-transfected cells treated with the same concentration of cisplatin was calculated by the Sidak’s post-test analysis, with * annotating a p-value <0.05. **(B, C)** A549cisR cell lysates were prepared either immediately at the end of the siRNAs (ATG5 or NT) transfections or two days following the end of the transfection (annotated as 2d). Western Blot analysis was performed for the detection of ATG5, LC3, p62 and α-tubulin proteins.

To make the determination that autophagy upregulation promoted cisplatin resistance in A549cisR cells, we evaluated the consequence of autophagy suppression on facilitating cisplatin cytotoxicity. As such, A549cisR cells were initially incubated for 24 hours with 25μM cisplatin prior to transfection with ATG5 siRNA or non-targeting (NT) siRNA for 2 days, followed by treatment with various cisplatin concentrations for an additional 24 or 48 hours, after which time, cell viability was measured. An effective ATG5 knockdown was confirmed by a dramatic reduction of ATG5 protein expression observed at 2 days after siRNA removal. This was paralleled by accumulation of p62 and diminished LC3 conversion, indicating a potent down-regulation of autophagy in our model (Fig 8B and 8C). By conducting a 2-way ANOVA test, we observed that ATG5 silencing negatively affected the viability of A549cisR cells treated with distinct cisplatin concentrations for 24 or 48 hours (Fig 8A). By conducting 1-way ANOVA analysis, we detected a statistically significant difference among the viability of ATG5-depleted A549cisR cells treated with different concentrations of cisplatin or 1%FBS at 24 hours. Indeed, cell viability was lower in cells treated with cisplatin versus 1%FBS. Specifically, the difference was statistically significant at cisplatin 25 μM, and close to significance at cisplatin 10 μM (p = 0.075). In contrast, no statistical difference in cell viability was detected in NT siRNA-transfected A549cisR cells exposed to various concentrations of cisplatin at 24 or 48 hours. Taken together, these results imply that autophagy inhibition through genetic suppression of ATG5 expression markedly enhances the cytotoxic activity of cisplatin against A549cisR cells. In addition, these observations indicate a crucial role for autophagy activation in the acquisition of the cisplatin resistance phenotype in lung cancer cells.

Of note, in a preliminary experiment, A549cisR cells were pretreated with siRNA ATG5 or NT followed by application of cisplatin in the presence or not of siRNAs. The cell viability was slightly higher in NT conditions as compared to ATG5 KD, although the difference was not statistically different (unshown data). This is the reason we decided to employ cisplatin pretreatment for 24 hours prior to suppressing autophagy with siRNA, and this is when we found the significant difference in cell viability between NT and ATG5 knockdown as mentioned above. This finding is intriguing, and it is presumable that cisplatin pre-exposure had selected the resistant cells that are the most dependent on autophagy to survive, thus rendering them more susceptible to autophagy suppression.

## Discussion

In this study we focused on (i) examining the apoptotic and autophagic response in lung cancer cells with acquired resistance to cisplatin (A549cisR), compared to parental cells (A549Pt), and (ii) exploring the capacity of chloroquine-induced LMP or specific autophagy blockade by ATG5 interference in restoring sensitivity to cisplatin. Hence, in our experimental design, we used parental A549 cell lines known to harbor *KRAS* mutation, and cisplatin resistant A549 cells selected following repetitive exposure of parental cells to incremental concentrations of cisplatin over time. A549cisR cells conceivably mimic what occurs in lung cancer patients who develop resistance to treatment after multiple cycles of platinum-based chemotherapy. The behavior of these cells was compared to their parental counterparts in an attempt to decipher some of the mechanisms responsible for drug resistance in NSCLC. We found that A549cisR cells displayed a blunted apoptotic response to cisplatin. Chloroquine potentiated cisplatin cytotoxicity against A549cisR cells in association with an LMP-mediated mechanism.

One of the major obstacles facing therapy of cancer is the development of refractoriness to chemotherapy, accounting for the poor clinical outcomes in many patients. A greater knowledge of the underlying escape mechanisms is crucial to developing novel therapeutic strategies for overcoming cisplatin resistance. Defect in the classical caspase-dependent apoptotic cell death often accounts for unresponsiveness to chemotherapy [16]. In this study, we initially determined that A549cisR cells exhibited a defective apoptotic response evidenced by a diminished activation of the early apoptotic marker, Annexin-V, along with the intrinsic apoptotic marker caspase 9 and caspase 3; these were reduced upon treatment with similar drug concentrations relative to parental cells. Surprisingly, we noticed a markedly greater expression of p53 in response to cisplatin in A549cisR cells, although Bak and Bax induction was lower, raising the question as to how important p53 activation is, to sufficiently induce lung cancer cell death.

Indeed, the role of p53 in determining sensitivity to cisplatin is controversial. Whereas some studies suggested p53 as an important mediator of cisplatin-induced apoptosis, other studies indicated that cisplatin-induced cell death occurs independent of p53 status [48, 52]. A relatively high level of wild type p53 has also been associated with cisplatin resistance [53].

In a study by Matsumoto *et al*, it was demonstrated in lung cancer cell lines that cisplatin induced apoptosis by activation of both p53-dependent Bak and p53-independent Bax expression [48]. In our model, the lack of Bak activation, despite an increase in p53, may be due to a defect pertaining to other mediators necessary for Bak activation. Notably, in the model applied by Matsumoto *et al*, Bak remained activated in response to cisplatin in shRNA-mediated p53 silenced cells, but to a lesser extent than shRNA control cells, implicating that factors other than p53 can also upregulate BAK.

Importantly, Matsumoto *et al* also reported that cisplatin-induced apoptosis of A549 cells was dependent on Bax activation, since silencing of Bax in cells lacking p53 was protective, suggesting that a lack of Bax activation could lead to cisplatin resistance. In concordance with this finding, we found that cisplatin failed to induce Bax expression in refractory lung cancer cells.

Chloroquine has been reported to synergize with chemotherapy in numerous cancer types. With few exceptions, the primary underlying mechanism of synergism described was autophagy inhibition [28, 30, 32, 33]. In contrast, Seitz *et al* described how chloroquine triggered apoptosis in concert with PI3 kinase inhibition in neuroblastoma cells, in an LMP-mediated fashion [27]. To our knowledge, the use of an LMP inducer to reverse cisplatin resistance in lung cancer was not previously reported. In our study, we provided direct evidence that chloroquine permeabilized the lysosomal membrane leading to cathepsin D release into the cytosol. Surprisingly, we discovered that the amount of LMP was more striking in refractory cells. This can be attributed to the increase in lysosome number in resistant cells, although a reduction in lysosomal membrane integrity in refractory cells cannot be ruled out. Previous studies showed that mefloquine, an antimalarial medication, was preferentially toxic against acute myeloid leukemia cells compared to normal hematopoietic cells, an effect that was linked to LMP. The impaired lysosomal membrane integrity and increased lysosomal mass demonstrated in leukemia cells may have influenced the selectivity of mefloquine against these particular cells [49].

Because chloroquine preferentially disrupted the lysosomal membrane of refractory cells, we exploited this vulnerable target to restore chemo-sensitivity. While its effect on A549cisR cell viability was marginal when used alone, chloroquine potentiated cisplatin-induced cell death in refractory cells. In addition, pre-treatment with E64, an inhibitor of lysosomal proteases, rescued cell viability. This is indicative of CQ-cisPt synergism that is, in part, promoted by the leakage of lysosomal proteases. However, cisplatin acted in cooperation with chloroquine at high concentrations, and this effect was not perceived at clinically relevant doses. Although, the degree of LMP was less pronounced with Chloroquine 25 μM, it effectively blocked autophagy in A549cisR cells at this concentration. Despite that, and unlike what we observed with siRNA ATG5, chloroquine 25 μM didn’t exhibit synergism with cisplatin against A549cisR cells. It is possible that due to other pleiotropic effect of chloroquine and lack of specificity, synergism was not observed. Surprisingly, although not statistically significant, E64 ameliorated the combination index of chloroquine/cisplatin combination at low doses of CQ (25 μM). It is plausible that E64 acted primarily by suppressing autophagy along with the low dose chloroquine, resulting to some degree in sensitizing the resistant cells to cisplatin. The dual role of chloroquine was previously described by Park *et al*, in a study on HCT15 colorectal cancer cells [54]. The authors found that, at low doses (10–20 μM), chloroquine acted as an autophagy inhibitor while at higher concentrations (40–160 μM), CQ triggered LMP through its detergent-like properties.

Similarly, we found no clear CQ-cisPt synergism in parental cells (data not shown). This lack of effect may be secondary to a diminished LMP, and a limited dependency on autophagy in parental cells. Notably, we discovered that A549cisR cells exhibit an increase in lysosomal number as measured by Cellomics imaging and LAMP-1 in cell lysates. This may explain why these cells are more prone to undergo LMP following exposure to chloroquine then their parental counterpart.

Another form of chemoresistance involves the sequestration of the chemotherapy drug into the lysosomal compartment followed by its exportation outside the cell through exocytosis [55]. LMP can result in the inability of lysosomes to accumulate and export toxic drugs, therefore favoring the intracellular retention of the drugs and thus enhancing cell death [16]. Since cisplatin is concentrated inside lysosomes, the greater lysosomal mass we observed in A549cisR cells may provide another explanation of the synergism between chloroquine and cisplatin in the resistant cells.

In an attempt to clarify the caspases involved in the drugs combination-induced cell death, we measured the activity of caspase 3, 8 and 9 in treated A549cisR and A549Pt cells. While chloroquine alone did not elicit caspase 9 activation, the enhancement of apoptosis induced CQ/CisPt occurred through the activation of the intrinsic apoptotic pathway, as manifested by an increase in caspase 9 activity. Conversely, the activation of the extrinsic pathway was only modest. Notably, the blockade of caspase activation protected A549cisR cells against cisplatin/chloroquine cytotoxicity. Although the observed protective effect was limited, this result implies that the synergism between the two drugs is partially but not entirely caspase-dependent. Our results are in agreement with accumulating evidence supporting the presence of cross-talk between lysosomal cell death and the mitochondrial death pathway. Boya *et al* reported that LMP induces cell death in a mitochondria-dependent fashion [20, 56]. Indeed, leaked cathepsin leads to mitochondria membrane permeabilization, release of cytochrome c and activation of caspase 9, the main effector of the intrinsic pathway [56]. Similarly, Erdal H *et al* demonstrated that this link between LMP and mitochondrial cell death is mediated by BAX [17]. While in A549cisR cells, BAK and BAX activation was reduced when cells were treated with cisplatin alone, it is possible that their residual activity may have been enough to synergize with the leaked cathepsin and activate the intrinsic pathway to a certain extent. Further mechanistic studies employing different specific caspase inhibitors will be useful in further analyzing the dependency of LMP on each caspase.

Chloroquine was previously reported to block autophagy, raising the question that this may have mediated, at least in part, the synergism between cisplatin and chloroquine in our current study. To address this question, we first compared the autophagic response upon cisplatin exposure in parental and resistant cells. We showed that cisplatin resistant cells exhibited a greater activation of autophagic flux at starvation, and following exposure to cisplatin compared to parental cells, using two different assays for autophagy detection. We particularly interrogated the dynamics of autophagosomal formation by assessing the turnover of LC3-II and cyto-ID loaded autophagosomes following treatment with lysosome inhibitors (E64 or chloroquine), a more reliable indicator of autophagic flux [47, 50]. We discovered that LC3-II and autophagosomes accumulation with cisplatin and lysosome inhibitor treatment occurred at a higher intensity in resistant cells. Furthermore, cisplatin increased p62 degradation much more in A549cisR cells. These observations clearly reveal that both cell lines differed in their capability to mount an autophagic response upon cisplatin treatment, with the autophagic flux being more induced in the resistant cells. Liu *et al* found that cisplatin activated autophagy in parental A549 cells; 3-MA, an autophagy inhibitor, markedly synergized with cisplatin to induce apoptosis [57]. Similarly, Ren *et al* observed an increased in autophagosome formation in A549cisR under serum starvation or radiation. The authors also reported that pharmacologic blockade of autophagy with 3-MA enhanced the apoptotic and cytotoxic effects of cisplatin in acquired resistant A549 cells [10]. In our study, we additionally demonstrated a preferential activation in the autophagic flux in A549cisR cells following exposure to cisplatin. Given the dual role and lack of specificity of 3-methyladenine in inhibiting autophagy, we reported a selective blockade of autophagosome formation by genetic silencing of ATG5 [51, 58]. We showed that sensitivity to cisplatin was potentiated by ATG5 knockdown, indicating that autophagy activation is associated with acquired cisplatin resistance in lung cancer.

This result was in line with previous findings, which suggested that chemotherapy might promote autophagy activation, and inhibition of autophagy may enhance the effects of chemotherapy [59].

Studies examining autophagy inhibition as a method to enhance the action of cisplatin have yielded contrasting results. For instance, Fukuda *et al* described the implication of autophagy in resistance to cisplatin in endometrial cells. This resistance was reversed by chloroquine, or ATG5/7 knock down [29]. Yao *et al* observed that blocking autophagy with chloroquine enhanced the therapeutic efficacy of MEK inhibitors in *KRAS* mutant lung cancer cells [25]. Conversely, in H1299 cells, a non-small-cell lung cancer cell line with innate resistance to cisplatin, monoplatin-induced cell death occurred via activation of autophagy, and independently of p53 and MAPK pathways [43]. Therefore, the role of autophagy in chemo-resistance appears to vary among different cancer and cell types. It is plausible that in acquired chemoresistance, autophagy is a mechanism of protection allowing for the selection of cells that are most likely to survive the effects of repetitive exposure of cytotoxic drugs.

In conclusion, our findings illustrate a rational for testing lysosomes targeting agents as a potential cisplatin sensitizer in refractory lung cancer. Screening for more potent LMP inducing compounds and designing a more selective delivery system to cancer cells through targeted nanoparticles may offer opportunities to develop novel effective therapeutic approaches for platinum-refractory lung cancer.

## Supporting information

S1 Fig Cell growth inhibition by cisplatin in parental and cisplatin-refractory A549 cell lines. A549Pt (A) and A549cisR (B) NSCLC cells were treated with varying concentrations of cisplatin (cisPt) for 72 hours. Cell viability was measured using the CellTiter-Blue^®^ assay. X-axis values represent log_10_ of each drug concentration. Y-axis represents the ratio of the fluorometric value at 72 hours divided by the value at 1 hour for each concentration and normalized to the negative control (1%FBS), which was set to 100%. Cisplatin IC_50_ concentrations were calculated using GraphPad Prism.
(TIFF)

S2 Fig A549cisR cells display a defective Bax and Bax, but not p53, response upon exposure to cisplatin. A549Pt and A549cisR cells were treated with cisplatin (10 μM) for the indicated time points and whole cell lysates were collected and immunoblotted for the indicated proteins (BAK, BAX, P53 and α-tubulin).
(TIFF)

S3 Fig A549cisR cells exhibit increased autophagic flux at baseline and following exposure to cisplatin under starvation condition, an effect that is less pronounced in parental cells. **(A)** A549Pt and A549cisR cells were plated in 24-well plates and grown for 24 hours. The following day, cells were pre-treated with or without E64 (10 μM) for 2 hours, followed by treatment in serum-free media for 16 hours. Levels of LC3-II and α-tubulin were determined by the densitometry of Western Blot (B) and (C) (condition cisplatin 0 μM). The bars in white represent the ratio of LC3-II levels adjusted to α-tubulin after treatment with E64 divided by the ratio value before treatment with E64. Black bars represent the ratio of LC3-II levels adjusted to α-tubulin after/prior E64 in case E64 has hypothetically no effect on LC3-II kinetics and is thus equal to 1. Graph bars represent Mean±SEM of three independent experiments. *p-value <0.05 white versus black bars. **(B, C)** A549Pt (B) and A549cisR cells (C) were plated in 24-well plates and grown for 24 hours, then treated overnight with different cisplatin concentrations in serum free media; whenever present, E64 (10 μM) was added for 2 hours prior to treatment with cisplatin. The following day, whole cell lysates were prepared and Western blot analysis was performed for the indicated proteins.
(TIFF)

## Abbreviations

A549cisR: cisplatin-refractory A549 cells
A549Pt: parental A549 cells
ALK: anaplastic lymphoma kinase
ATG: autophagy-related protein
Bak: Bcl-2 homologous antagonist killer
Bax: Bcl-2-associated X protein
CisPt: cisplatin
CQ: chloroquine
DAPI: 4’, 6-diamidino-2-phenylindole
EGFR: epidermal growth factor receptor
FBS: fetal bovine serum
FITC: Fluorescein isothiocyanate
IF: immunofluorescence
KD: knockdown
*KRAS*: Kirsten rat sarcoma viral oncogene homolog
LAMP-1: lysosomal-associated membrane protein 1
LC3: microtubule associated protein 1 light chain 3
LMP: Lysosomal Membrane Permeabilization
NSCLC: non-small-cell lung cancer
NT: Non-targeting
PD-L1: programmed death-ligand 1
PI3K: phosphatidylinositol-3 kinase
PI: Propidium iodide
SEM: standard error of the mean
SF: serum-free
STP: staurosporine
TKI: tyrosine kinase inhibitor
WB: Western Blot
