# Correction: Modulating lysosomal function through lysosome membrane permeabilization or autophagy suppression restores sensitivity to cisplatin in refractory non-small-cell lung cancer cells

**DOI:** 10.1371/journal.pone.0197016

**Published:** 2018-05-02

**Authors:** Magdalena Circu, James Cardelli, Martin P. Barr, Kenneth O’Byrne, Glenn Mills, Hazem El-Osta

There is an error in the third author’s name. The correct name is Martin P. Barr. The correct citation is: Circu M, Cardelli J, Barr MP, O’Byrne K, Mills G, El-Osta H (2017) Modulating lysosomal function through lysosome membrane permeabilization or autophagy suppression restores sensitivity to cisplatin in refractory non-small-cell lung cancer cells. PLoS ONE 12(9): e0184922. https://doi.org/10.1371/journal.pone.0184922
