# Supplementary figures and images for: Modulating lysosomal function through lysosome membrane permeabilization or autophagy suppression restores sensitivity to cisplatin in refractory non-small-cell lung cancer cells
Source: PLoS One. 2017 Sep 25;12(9):e0184922. doi: 10.1371/journal.pone.0184922 (PMC5612465; doi:10.1371/journal.pone.0184922)

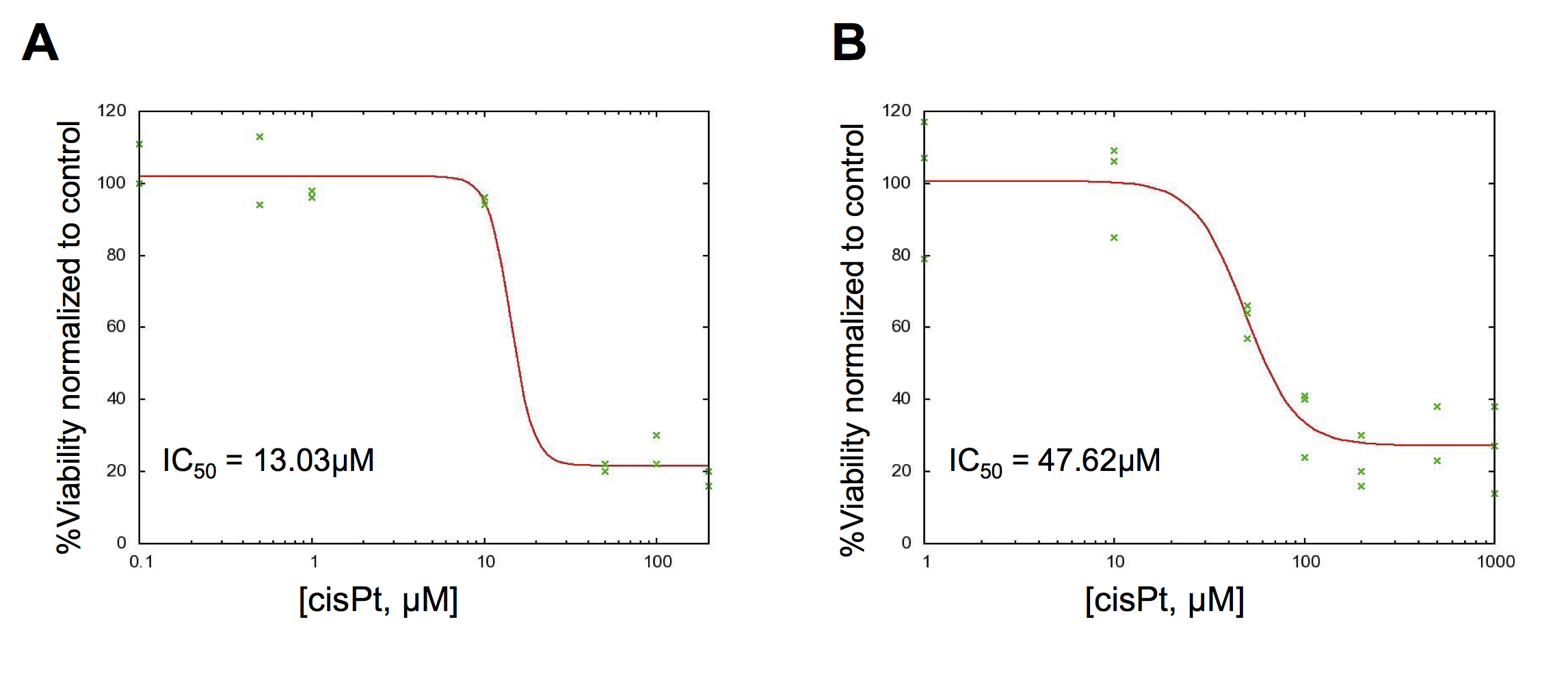

Supplement: S1 Fig — A549Pt (A) and A549cisR (B) NSCLC cells were treated with varying concentrations of cisplatin (cisPt) for 72 hours. Cell viability was measured using the CellTiter-Blue® assay. X-axis values represent log10 of each drug concentration. Y-axis represents the ratio of the fluorometric value at 72 hours divided by the value at 1 hour for each concentration and normalized to the negative control (1%FBS), which was set to 100%. Cisplatin IC50 concentrations were calculated using GraphPad Prism. (TIFF) [file pone.0184922.s001.tiff]

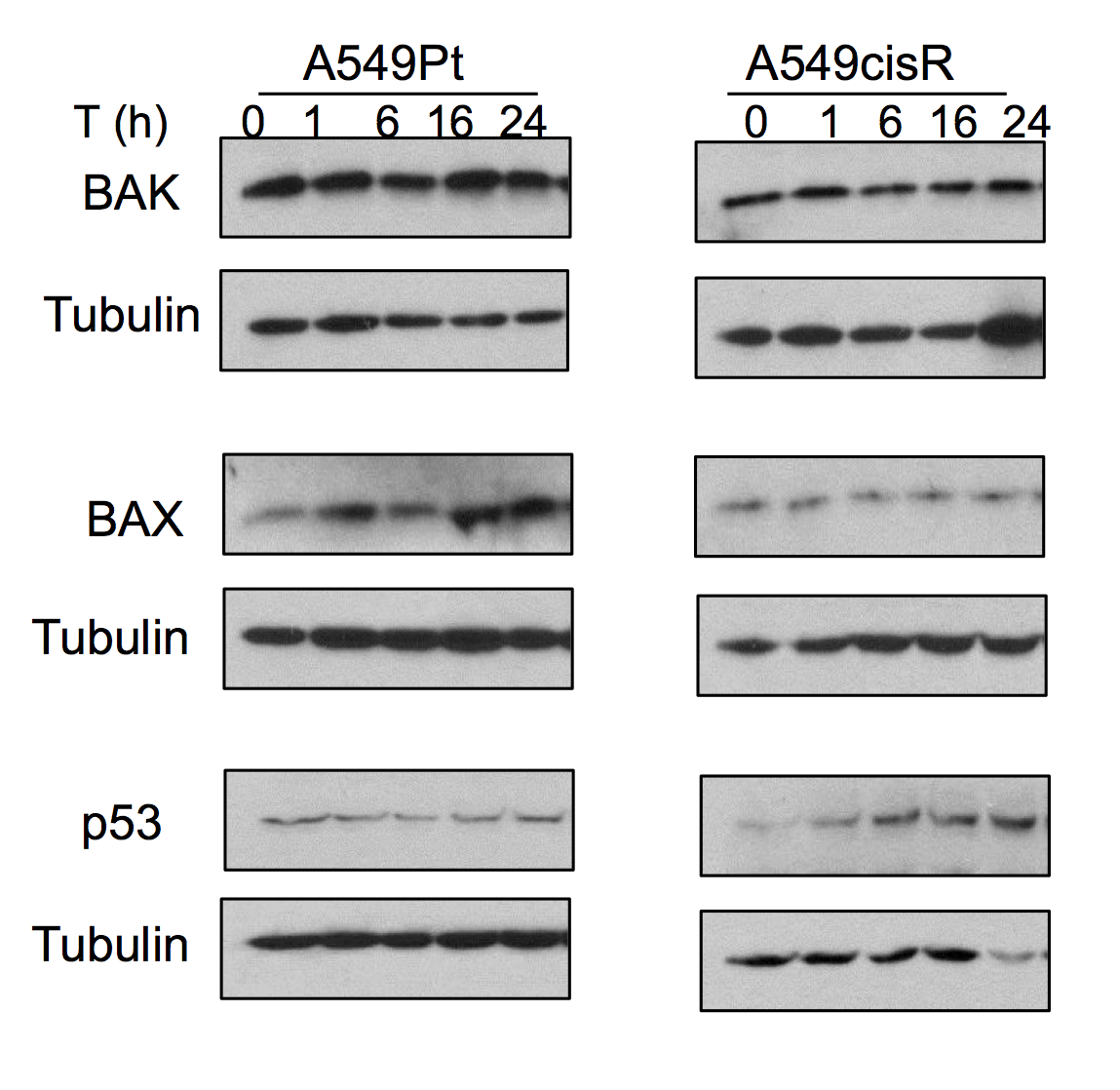

Supplement: S2 Fig — A549Pt and A549cisR cells were treated with cisplatin (10 μM) for the indicated time points and whole cell lysates were collected and immunoblotted for the indicated proteins (BAK, BAX, P53 and α-tubulin). (TIFF) [file pone.0184922.s002.tiff]

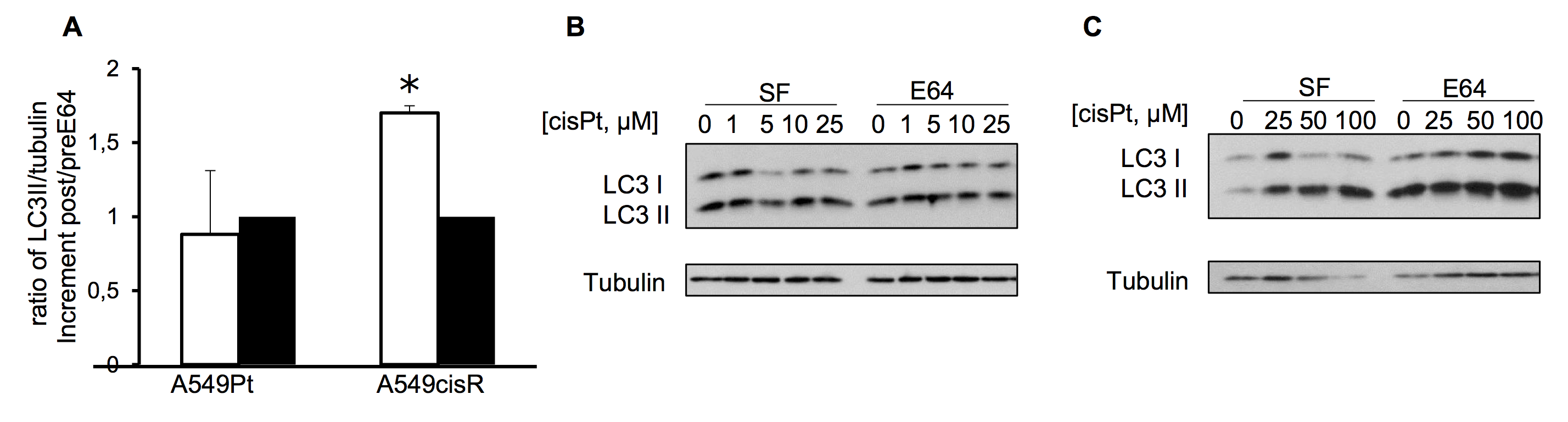

Supplement: S3 Fig — (A) A549Pt and A549cisR cells were plated in 24-well plates and grown for 24 hours. The following day, cells were pre-treated with or without E64 (10 μM) for 2 hours, followed by treatment in serum-free media for 16 hours. Levels of LC3-II and α-tubulin were determined by the densitometry of Western Blot (B) and (C) (condition cisplatin 0 μM). The bars in white represent the ratio of LC3-II levels adjusted to α-tubulin after treatment with E64 divided by the ratio value before treatment with E64. Black bars represent the ratio of LC3-II levels adjusted to α-tubulin after/prior E64 in case E64 has hypothetically no effect on LC3-II kinetics and is thus equal to 1. Graph bars represent Mean±SEM of three independent experiments. *p-value <0.05 white versus black bars. (B, C) A549Pt (B) and A549cisR cells (C) were plated in 24-well plates and grown for 24 hours, then treated overnight with different cisplatin concentrations in serum free media; whenever present, E64 (10 μM) was added for 2 hours prior to treatment with cisplatin. The following day, whole cell lysates were prepared and Western blot analysis was performed for the indicated proteins. (TIFF) [file pone.0184922.s003.tiff]
